# Supplementary material for: Are carbon emissions trading and green financial instruments synergistic? -Comprehensive quantitative research based on content analysis
Source: PLoS One. 2024 Mar 7;19(3):e0298601. doi: 10.1371/journal.pone.0298601 (PMC10919601; doi:10.1371/journal.pone.0298601)
Supplement: S1 Data — (ZIP) [file pone.0298601.s001.zip › Data/地级市Z_F工作报告.zip!/║╙─╧/╜╣╫≈╩╨/└┤╫╘╣1⁄2╓┌║┼í╛┬φ┐╦╩2╛▌═°í┐_2015.docx]

各位代表：

现在，我代表市人民政府，向大会报告工作，请予审议，并请各位政协委员和列席人员提出意见。

一、2014年工作回顾

2014年是本届政府履职的第一年。一年来，面对严峻复杂的经济形势，在省委、省政府和市委的正确领导下，在市人大常委会、市政协的监督支持下，市政府深入学习贯彻党的十八大和十八届三中、四中全会精神，深入贯彻落实习近平总书记系列重要讲话精神，深入开展党的群众路线教育实践活动，凝心聚力、转型攻坚、争创一流、绿色发展，大力实施工业强市、科技强市战略，强力推进经济转型示范市和美丽焦作建设，经济社会保持了平稳较快发展。全市生产总值1846亿元，增长8.8%；规模以上工业增加值1073亿元，增长10.7%；一般公共预算收入106亿元，增长8.5%；固定资产投资1624亿元，增长18.2%；社会消费品零售总额555亿元，增长12.2%；城镇居民人均可支配收入23977元、农民人均纯收入12518元，分别增长8.7%、10.1%。

（一）改革开放全面深化。把改革作为科学发展的根本动力，摆在首要位置，强力推进。大力简政放权，行政审批制度改革取得重大突破，我市成为全省行政审批标准化试点市。市本级行政审批服务由718项压减到253项，压减量居全省首位，压减率达65%；市本级建设工程类项目审批时限由300个工作日压缩到40个工作日；建立首席代表负责、并联审批、全程代办等新机制，在全省首家开通网上服务大厅，简单事项实现即收即办，一般事项一审一核不超过5个工作日，新注册企业“四证一章”办理不超过2个工作日；全社会投资创业积极性得到释放，新增市场主体2万余户。建立健全财税收入征管联动机制，社会综合治税成效显著，增收3.8亿元。新增国家义务教育发展基本均衡县市区2个，总数达到5个，我市成为全省新一轮教育信息化试点。县级公立医院综合改革持续深化，医疗联合体建设全面启动。农村、户籍、文化等改革都取得阶段性成果。

坚持激活内力与借助外力相结合，深入开展“对外开放提升年”活动，成功举办焦作·香港现代服务业招商推介等活动，大力开放招商，积极承接产业转移，加快发展开放型经济。引进省外境内项目394个，居全省首位；总投资769亿元，实际到位资金498亿元，居全省第三位。进出口总额24.6亿美元，居全省第二位，其中出口17亿美元，居全省第三位。

（二）十大建设成效显著。强化领导分包、观摩督导等措施，省、市重点项目加快推进，一批十大建设项目竣工投运。城市水系防洪、补源、生态、景观等功能已经显现，南水北调总干渠全线通水，引黄入焦干渠、大沙河带状湿地主体工程完工，新增水面7000亩；郑焦城际铁路正在验收；北部山区生态治理恢复和南太行生态景观带建设加快，我市成为全省首批生态文明先行示范市；海关监管场所正式运营，检验检疫查验场所即将投用，德众保税物流中心通过国家验收；产业集聚区21个投资10亿元以上项目竣工投产；财税信息共享平台建设顺利推进，“数字焦作”地理空间框架工程通过国家验收；太极体育中心建成投用；龙源电厂一号机组正式运行，焦作电厂异地扩建一号机组、龙源电厂二号机组即将试运行；武云高速等项目快速推进；职业技术学校新校区招生人数居全省同类学校第一位。

（三）工业经济稳定增长。坚持工业主导地位，强力实施工业强市战略，突出抓转型、调结构、保运行，工业经济保持平稳增长态势。转型步伐加快。深入开展“工业转型升级攻坚年”活动，出台加快城区工业转型发展意见，实施“330”企业培育工程，100个投资超亿元转型升级重点项目完成投资222亿元，大江重工中高端液压支架、千年冷冻高效节能制冷系统等32个项目竣工投产，规模以上工业企业总数达1186家。发展载体夯实。聚焦项目建设、聚集优质资源、聚合政策支持，示范区和9个省级产业集聚区承载能力有效提升，我市在全省重点项目暨产业集聚区建设观摩点评活动中获豫北组第一名；入选省“十强”“十快”产业集聚区3家、星级产业集聚区5家，均居全省第二位。融资服务提升。整合工业投融资平台，设立政府引导基金，搭建应急转贷平台，建立小额贷款风险补偿机制，为7家企业提供5900万元应急转贷资金支持，为150家企业担保贷款17亿元，缓解了中小企业融资难题。节能减排扎实推进。加快国家首批资源综合利用“双百”工程示范基地建设，强力实施蓝天、清水等工程，节能减排完成省定目标任务，累计争取国家资源枯竭城市资金23.8亿元。

（四）创新能力不断增强。坚持实施科技强市战略，完善创新体制机制，突出财政杠杆撬动，加快发展动力转换，创新驱动效应初显。科技扶持力度进一步加大，市本级科技投入又翻一番，总量居全省第三位。继续实施创新券、科技贷款贴息等制度。设立1000万元知识产权质押贷款风险补偿金，撬动银行10亿元授信支持。创新平台建设加快，新增院士工作站7家，总数达19家，居全省第三位；新增省级企业技术中心4家，总数达81家；新增省级工程技术中心4家，总数达54家。中关村科技成果产业化（焦作）基地、中科院科技成果转化（焦作）中心落地项目10个，引进北京化工大学等科技创新团队9个，焦作科技大市场即将开业运营。新增国家高新技术企业8家，总数达42家，高技术产业增加值增长25.9%，高于规模以上工业15.2个百分点。

（五）现代农业快速发展。坚持稳粮、提质、增效，新建高标准粮田27万亩，粮食总产实现“十一连增”，夏粮单产保持全省第一。农业产业化集群加快发展，龙头企业达170家。主要粮食综合机械化水平保持在96%以上，居全省前列。农民专业合作社达3826家，覆盖率达49%。农村土地流转率达51.7%，高于全省15.7个百分点。农业科技创新力度加大，农技推广服务体系进一步健全。成功创建“四大怀药”国家级出口食品农产品质量安全示范区，成功创建国家级农产品品牌3个。新完成一批中小河流治理和水库除险加固工程。深入开展国家森林城市创建工作，完成森林资源培育21万亩，森林覆盖率达28.5%。

（六）服务业发展亮点纷呈。把服务业作为弥补产业短板、推动转型升级的主攻方向，抢抓发展机遇，瞄准产业动向，加快培育经济新增点。新业态发展取得突破。组建高规格团队大力招商，市电子商务产业园开工建设，浪潮集团、银江科技、阿里巴巴等27家电商落户我市，电子商务成为服务业发展的新亮点。金融业活力持续增强。完善考核激励机制，金融机构各项贷款余额862亿元，增长10.5%。大力发展直接融资，全市利用资本市场融资151亿元。加快推进企业进入多层次资本市场，宇建科技等3家企业在“新三版”成功挂牌。海通证券等3家券商营业部开业运营。我市被评为优秀金融生态省辖市。文化旅游业保持繁荣。云台山景区成为全省首家全国服务标准化示范单位，我市成功创建国家级旅游服务综合标准化示范市，被世界旅游组织列为可持续发展监测点。旅游综合收入达262亿元，增长12%。商贸物流业快速发展。新河商务中心区规划建设全面启动，鸿运国际商城开工建设，王府井百货开业运营。积极融入“一带一路”战略，我市成为丝绸之路经济带物流联动发展合作联盟成员城市。

（七）城乡建设加快推进。出台新型城镇化三年实施方案，强化经营城市理念，积极探索创新，推动新型城镇化科学发展。城市功能进一步完善。新开工保障房8409套，基本建成8304套。新市民社区试点开工项目6个，建成住宅6996套。新改建城市道路35公里、供水管网41公里、燃气管网98公里，建成游园6个。购置新能源公交车30台，我市成为首批省级公交优先示范市创建城市。发展空间进一步拓展。总投资38亿元的迎宾路南延及焦作黄河大桥项目实现重大突破，即将开工建设。总投资37亿元的南太行绕城高速项目前期工作进展顺利，东海大道、南外环建设加快，城市大外环框架加快构建。14条省道规划全部获批，新增干线公路里程510公里。城市经营管理进一步加强。引入社会资本参与城市建设，城中村改造前期工作全面启动，东区热电机组配套供热管网建成投用，龙源电厂配套供热管网即将建成。强力开展“双违”集中整治，严格土地收储交易管理。积极推进小型汽车号牌等公共资源经营。深入开展背街小巷、户外广告等专项整治，城市管理水平有效提升。新农村建设进一步加快。扎实推进美丽乡村建设，深入开展城乡环境卫生治理，建立健全保洁长效机制。新改建农村公路306公里，解决了22.8万名农村群众和师生的安全饮水问题。

（八）民生保障持续提升。在支出压力增大的情况下，财政民生支出145亿元，增支12亿元。省、市十项重点民生工程全面完成，人民群众得到更多实惠。社会保障更加有力。在全省率先实行城镇医保按比例报销上不封顶制度，惠及95万名参保群众；在全省率先建立商业保险医疗救助制度，为15万名城乡困难群众购买商业保险服务；在全省率先完善城乡困难群众临时救助制度，救助9334人次；全面启动保险机构参与的新农合大病保险制度，260万名农村群众从中受益。城乡低保标准和城乡居民、企业退休人员养老保险待遇进一步提高。残疾人保障体系不断健全。大力发展养老服务业，新增养老床位3000余张。出台就业创业扶持政策，城镇新增就业8.8万人，农村劳动力新增转移就业3.3万人，我市成为首批全国公共就业人才服务标准化试点市。住房公积金创新受托银行管理模式，增值收益突破亿元大关。社会事业加快发展。黄河交通学院成功升本，新城小学、丰泽园小学、四医院综合病房楼等一批中小学幼儿园、医院项目和文化惠民工程推进加快。我市顺利通过国家二类城市语言文字评估验收。成功举办省十二届运动会暨首届全民健身大会。“单独二孩”政策顺利实施，我市成为首批全省人口均衡型社会综合配套改革示范市。和谐稳定大局有效巩固。加强社会治安综合治理，加强应急管理工作，加大普法宣传教育力度，积极化解信访积案，强化安全生产监管，深化食品药品安全专项整治，严厉打击和处置非法集资，做好民族宗教工作，发展志愿者事业，推动军民融合深度发展，驻焦部队和民兵预备役为地方发展作出新贡献，社会大局保持和谐稳定。

（九）自身建设得到加强。扎实开展党的群众路线教育实践活动，强力整治“四风”突出问题，工作作风有效改进，行政效能不断提升。稳步推进政府机构和事业单位分类改革。加大政务公开力度，市直部门预算及“三公”经费预算全部公开。厉行勤俭节约，“三公”经费下降32%。在全省首家开展公共资源交易改革，电子交易系统全面启动。大力推进依法行政，自觉接受人大法律监督、工作监督和政协民主监督，强化行政监察，严格审计监督。统计调查、外事侨务、档案史志、台湾事务、人防、广电、气象、地震等工作都取得了新的成绩。

各位代表！回顾一年来的工作，在经济下行压力大、困难挑战多的情况下，我市经济社会发展取得这些成绩来之不易。这是市委正确领导的结果，是市人大常委会、市政协、焦作军分区大力支持的结果，是全市人民共同奋斗的结果，在此，我代表市人民政府，表示衷心的感谢！

我们也清醒地认识到，经济社会发展、政府工作和自身建设还存在一些问题。一是经济下行压力较大。企业生产经营困难和问题增多，投资增速持续回落，财政收入增速放缓，一些潜在风险逐步显现。二是转型升级任务艰巨。战略性新兴产业尚未形成有效支撑，服务业占比偏低，高新技术产业规模偏小，经济新增点较少，发展质量效益不高。三是改革创新仍需攻坚。市场经济、经营城市等理念还没有落实到经济社会发展的全过程、各个环节，制约发展的传统思想藩篱依然存在，一些体制机制障碍仍未破除，创新驱动和内生增长动力仍显不足。四是民生薄弱环节较多。就业、就学、就医、住房等公共服务与群众期盼还有差距，交通拥堵、环境污染等群众反映强烈的问题需下更大功夫解决。五是作风建设还需加强。工作作风、能力素质与经济发展新常态要求还不适应，担当意识不强、推进落实不力、效率效能不高，一些领域还存在腐败现象。对此，我们一定采取有力措施，扎实有效解决，决不辜负人民期望！

二、2015年工作总体要求和主要目标

2015年是全面深化改革的关键之年，是全面推进依法治国的开局之年，也是全面完成“十二五”规划的收官之年。当前，经济发展进入新常态，我市仍处在加快转型发展的重要战略机遇期，新一轮产业革命正孕育兴起，我们更加注重新技术、新业态、新商业模式引领，推动电子商务、智能制造、工业机器人、新能源汽车等产业蓬勃发展，经济新增点不断涌现，必将为我市抢得先机、占领转型升级制高点奠定坚实基础；我们坚定不移地通过全面深化改革破除体制机制障碍，以思想观念创新带动工作方式、管理方式创新，必将为经济社会发展增添新动力、激发新活力；我们积极探索实践，科学推进新型城镇化，促进“四化”同步发展，必将持续拉动投资、释放需求、扩大消费，为经济平稳健康发展增添新动能；我们充分发挥现有一大批行业龙头企业和高素质产业工人的优势，不断完善示范区、产业集聚区等科学发展载体，必将更快更好承接产业转移，做大做强实体经济；我们夯实现代交通体系支撑，郑焦城际铁路即将通车，迎宾路南延及焦作黄河大桥即将开工，郑太铁路客运专线项目前期工作进展顺利，区位优势更加凸显，必将为我们加快焦郑融合、与晋东南合作拓展广阔空间；我们主动融入“一带一路”国家战略新布局，为用好“两个市场”“两种资源”开辟新的大通道，必将促进我市开放型经济快速发展，构筑开放新高地；我们持续加强生态文明建设，城市水系建设已见成效，林业生态网络加快构建，努力让森林走进城市、让城市拥抱森林，打造宜居、宜业、宜游的花园城市，必将为增进人民福祉、增强可持续发展能力创造良好条件；我们坚持以行政审批制度改革为突破口，大力简政放权，提升效率效能，必将为经济社会发展创造好环境、打造新引擎。只要我们紧紧抓住机遇，坚定信心、主动作为、乘势而上，就一定能把加快发展的正能量激发出来，把转型发展的巨大潜力释放出来，建设美丽焦作、全面建成小康社会的目标就一定能够实现！

今年政府工作总体要求是：以邓小平理论、“三个代表”重要思想、科学发展观为指导，全面贯彻党的十八大和十八届三中、四中全会精神，深入贯彻习近平总书记系列重要讲话精神，认真落实省委、省政府和市委决策部署，坚持“凝心聚力、转型攻坚、争创一流、绿色发展”的要求和“一个战略、四项重点、十大建设”的思路不动摇，主动适应经济发展新常态，坚持调中求进、改中激活、转中促好、变中取胜，突出调整经济结构，突出深化改革开放，突出培育新增长点，突出保障改善民生，突出创新驱动，突出风险防控，着力提高经济发展质量和效益，促进经济平稳健康发展和社会和谐稳定，全面加快经济转型示范市和美丽焦作建设。

经济社会发展主要目标是：生产总值增长8.5%；一般公共预算收入增长9%；固定资产投资增长19%；社会消费品零售总额增长13%；进出口总值增长9%；城镇新增就业6万人以上；城镇居民人均可支配收入增长9%，农民人均纯收入增长9%；节能减排和环境质量改善完成省下达任务。

三、2015年重点工作

今年的任务艰巨繁重，重点做好九个方面工作：

（一）着力深化改革开放，增强发展动力活力。聚焦制约经济社会发展的突出障碍，深入推进重要领域和关键环节改革，进一步激发市场活力、增强内生动力、释放改革红利，以改革提效率、以改革促发展。深化行政审批制度改革。加快推进全省行政审批标准化试点市建设，大力简政放权，继续取消和下放行政审批事项，全部取消非行政许可审批事项，全面公开权力清单、责任清单，全面推进网上预受理、预审查，全面运行并联审批制度，确保在优化审批环节、压缩审批时限上取得新突破，创造焦作效率，打造焦作品牌，走在全省前列。深化财税体制改革。完善政府预算体系，加强预算执行管理，建立全面规范、公开透明的现代预算制度。规范政府债务管理，积极防范和化解债务风险。坚持税收法定原则，全面规范税收优惠政策。深入开展综合治税，提升财税精细化管理水平。深化教育医疗改革。加快推进国家义务教育均衡发展试点市创建，加快发展现代职业教育，大力发展民办教育。积极推动医疗联合体建设，深入推进县级公立医院、医保支付方式、全科医生执业方式和服务模式改革，探索开展城市公立医院改革。深化农村改革。坚持土地公有制性质不改变、耕地红线不突破、农民利益不受损三条底线，开展农村经营主体金融授信、农业大灾风险补偿等改革工作，积极争取农村土地征收、集体经营性建设用地入市、宅基地制度改革等试点，探索增加农民财产性收入的渠道。做好现代农业综合配套改革省级试验区工作。同时，加快推进政府机构、文化体制、公共资源交易、交通行政执法体制等各项改革。

积极融入“一带一路”战略，高水平引进来，大规模走出去，以开放带动发展、活跃全局。充分发挥招商优势。尽快转变比拼优惠政策的招商模式，更加注重集群式、链条式产业招商，更加注重发挥我市工业基础坚实、集聚效应明显、配套能力突出等比较优势，努力在承接产业转移的竞争中抢占先机。积极创新招商方式。突出企业招商主体地位，紧盯国内外500强和行业龙头，大力开展精准招商、以商招商、中介招商，重点推进与中车集团等合作取得实质成果。完善提升开放平台。深化关检合作，提高通关效率，降低企业成本。加强出口基地和出口产品质量安全示范区建设，争取示范区成功创建省级服务外包示范园区，推动产业集聚区申报省级出口基地。不断扩大开放领域。放宽市场准入，加快城乡基础设施、服务业、社会事业、农业等领域开放，积极推行政府与社会资本合作模式，充分发挥市场机制作用，引进更多社会力量、民间资本，用市场杠杆撬动资金、人才等要素，促进资源优化配置，提升开放型经济规模和效益。

（二）着力加快十大建设，持续增强发展后劲。把项目建设作为重中之重，以培育经济新增点、培植新的税源财源、调优经济结构为主线，持之以恒抓好项目建设。强化十大建设带动。城市水系方面，全面启动新河治理，加快灵泉湖、圆融水库等工程建设，完成大沙河带状湿地一期等工程。铁路方面，加快推进郑太铁路客运专线焦作段前期工作。生态方面，加快方山等矿山地质环境治理，推进南太行国家矿山公园等工程。示范区和产业集聚区方面，新开工10亿元以上项目24个，完成投资120亿元以上。信息化方面，加快建设智慧焦作，推进焦作云计算中心平台等建设。能源方面，龙源电厂、焦作电厂异地扩建工程全面建成投产，开工建设丹河电厂上大压小异地扩建工程。公路方面，完成武云高速主体工程，开工南太行绕城高速等项目。职教园区方面，加快推进市技师学院新校区规划建设。电子商务暨“两区”方面，完善市电子商务产业园一期配套服务功能，开工建设二期；全面开展新河商务中心区土地收储、征迁安置等工作，全面开工配套道路、高压线改造等基础设施项目；大力推进鸿运国际商城等项目，加快特色商业区建设。健康服务业方面，开工建设市福利院老年公寓楼、残疾人康复托养服务中心等项目。强化重点项目支撑。今年安排市重点项目198个，总投资1293亿元，年度完成投资412亿元。集聚要素资源，加快建设步伐，早日建成投产，用项目建设成效促进财税增收、提高发展质量。强化项目谋划储备。科学编制“十三五”规划，谋划好全局性、长远性、战略性的重大项目，力争更多项目进入国家、省级盘子，为经济社会发展备足后劲。

（三）着力新型工业化发展，加快工业转型步伐。坚持工业强市战略，以转型升级为主线，突出抓好十大产业，引领产业产品向智能化、高端化、终端化发展，抢占新一轮产业转型升级制高点。加快十大产业转型升级，推动产品结构向高端迈进。以城区工业十大振兴项目为重点，加快推进100个重大转型升级项目建设，完成投资176亿元。围绕国家新型工业化示范基地建设，重点推进河南正旭航空航天3D打印焦作基地、河南新力克喷浆机器人等项目；围绕壮大国家汽车零部件特色产业基地规模，重点推进风神轮胎高性能乘用子午胎、中内配轴瓦等项目；围绕打造国家循环经济化工基地，重点推进佰利联氯化法钛白粉等项目；围绕加快新能源产业发展，积极申报新能源汽车试点城市，重点推进多氟多能量型动力锂电池等项目，大力支持多氟多发展新能源汽车，立雄心壮志、拼最大干劲，圆焦作新能源汽车整车梦；围绕加快食品工业发展，重点推进蒙牛乳业纯甄酸奶等项目；围绕拉长铝工业链条，重点推进奋安铝业新型高端铝型材等项目；围绕生物医药、新材料、节能环保、电子信息等新兴产业发展，重点推进中国铁路通信信号焦作产业园、健康元医药中间体等项目。大力发展智能制造业，加快传统制造转型升级。顺应新一轮技术革命趋势，把发展智能制造作为工业转型升级的战略重点，以应用为核心、市场为导向，依托我市工程机械、汽车及零部件、矿山机械、造纸机械等传统制造业，推进与大连理工大学等知名高校和科研院所合作，瞄准3D打印设备、智能机器人、智能节能环保装备、高档数控机床、自动化成套设备等领域，尽快落地一批项目，全力发展智能制造业，努力打造全省乃至全国的智能制造基地。加快示范区和产业集聚区建设，筑牢科学发展载体。抓住全国土地利用总体规划调整完善的重大机遇，科学调整全市土地利用总体规划，大力拓展示范区发展空间，推动示范区在构建现代产业体系和现代城乡体系、公共服务均等化、生态文明建设等方面先行先试，着力打造产业融合、城乡一体、统筹发展的复合型功能区，努力走在全省示范区前列。实施产业集聚区提升工程，提高产业集群、配套服务、节约集约、产城互动发展水平，9个省级产业集聚区大力争创全省十强十快十先进。保障工业经济运行，优化企业发展环境。强化要素保障，提供高效服务，保运行、促转型。加快两化融合，培育市级以上两化融合示范企业12家。推进名牌带动、标准引领和质量创新，提升企业核心竞争力。推动企业进入多层次资本市场，加强上市（挂牌）后备企业指导服务，让更多企业通过资本市场做大做强。

（四）着力实施科技强市，打造创新驱动引擎。以创建国家创新型试点城市和知识产权试点城市为载体，健全科技创新体制机制，提高科技投入使用绩效，加快构建焦作特色自主创新体系，进一步提升科技引领转型、创新驱动发展的能力。大力推进协同创新。引导企业把创新重点放在开放合作上，加强与行业龙头、高等院校、科研院所和国家级自主创新示范区对接，深化与中科院、中关村等合作，引进一批高科技成果，落地一批高科技项目，组建一批创新战略联盟，加快科技前沿技术和先进适用成果转化应用，提升焦作产业整体装备水平和工艺技术水平。积极搭建创新平台。新增一批省级以上高新技术企业，新建一批省级以上研发中心和院士工作站。与大连理工大学合作组建焦作装备制造业公共研发中心，加快河南理工大学科技园建设。运营好焦作科技大市场，打造全省科技资源集聚的新平台、科技成果转移转化的新高地。健全完善体制机制。大力发展科技金融，完善金融支持科技创新政策，出台扶持科技型中小企业融资办法，实施知识产权质押贷款制度，探索成立服务科技创新的银行专门机构。整合市县两级财政科技资金，研究制定统筹使用办法，探索利益共享、风险共担新机制；继续实施创新券、贷款贴息等制度，最大限度发挥财政资金杠杆作用和放大效应，为高科技企业插上腾飞翅膀，让我市转型发展源于科技创新、兴于科技金融、成于创新驱动，加快打造焦作经济升级版。

（五）着力推进新型城镇化，加快城乡一体化发展。坚持以人为核心，坚持以农业转移人口市民化为重点，坚持“一基本两牵动三保障”，更加注重经营城市，更加注重运用市场机制，科学推进新型城镇化发展。优化空间布局。开通郑焦城际铁路，加快迎宾路南延及焦作黄河大桥项目建设，打通焦作至郑州的快速通道，主动向以郑州为中心的组合型大都市区融合发展。加快焦修博武一体化，推动中心城市组团式发展。加强县城建设发展，着力建好吸纳农业转移人口的主载体。强化政策保障。落实户籍制度改革政策，研究制定就业、教育、医疗、社会保障等配套改革措施。切实保障进城农民的土地承包经营权、农户宅基地用益物权和农民集体经济收益分配权，让农民带着权益进城、带着保障落户。增强承载能力。全面推进新市民社区试点建设，建成3个试点社区。开工建设保障性住房5707套，基本建成7396套。加快城中村和棚户区改造，启动南水北调两侧绿化带及配套设施建设。加快中心城市大外环建设，实施民主路等主干道延伸工程，完善示范区路网体系，改造一批瓶颈路，打通一批断头路。全面加快城市污水管网建设，实现中心城区全覆盖。改造供水管网30公里、燃气管网50公里，新增供热面积500万平方米。加快推进世界银行贷款公交项目建设，再更新一大批新能源公交车，下大力气打造省级公交优先示范市。规范经营城市。继续严厉打击“双违”，规范管理土地市场，严格收储出让制度，深入推进道路桥梁冠名、路牌广告等公共资源经营，增强政府科学配置资源、推动城市可持续发展能力。深入开展市容市貌专项整治，扎实推进城乡接合部环境卫生治理。拓宽筹资渠道。设立新型城镇化发展基金，积极争取省基金支持；利用投融资平台进行市场化运作，推进市政债券发行；采取股权融资、特许经营等方式，吸引更多社会资本参与基础设施建设，积极构建市场化、可持续的新型城镇化建设融资新机制。

（六）着力发展现代服务业，调整优化产业结构。把发展现代服务业作为稳增长、调结构的突破口，瞄准新业态、新模式，抢占先机、跨越发展，努力把现代服务业的巨大潜力转化为现实生产力。以新型业态为产业导向，培育经济新增点。大力发展电子商务，围绕市电子商务产业园、新河商务中心区和示范区、焦西、武陟等电商物流园建设，完成电子商务发展规划，优化电子商务发展布局。大力引进电商龙头、现代物流和互联网金融、物流金融企业，全力支持百度、新浪、中青网、北斗星通、一亩田、浪潮集团等区域总部和运营中心建设，强力推进阿里巴巴产业带、韩国跨境电商平台、慧聪网、58同城、360搜索、银江科技、淘宝大学等项目尽快入驻运营，加强与京东、当当、中国兵工、中电科、南方物流等公司的战略合作，积极创建省级、国家级电子商务示范基地，做大做强电子商务产业，力争通过3年努力，使我市电子商务率先在全省取得重大突破、实现跨越发展。以商业模式创新为重要途径，激活消费需求。实施商业模式创新示范工程，探索形式多样的新兴商业模式，引导和鼓励企业线上线下连通、虚拟实体结合，利用电子商务进行市场营销，强力推进电子商务进农村进社区，积极发展直营店和乡村连锁商业中心，加快建设现代城市综合体，发展商务楼宇经济，在满足个性化、多样化消费需求上赢得主动，进一步释放城乡消费潜力。以金融创新为核心抓手，打通实体经济血脉。构建企业-政府-银行“铁三角”，强化市县两级政府信用担保体系建设，提高政府性中小企业担保公司担保能力；建立装备制造、节能环保等产业基金，加快建设政府引导基金体系；深化政府、银行、保险三方合作，完善小额贷款风险补偿机制；加强应急转贷平台建设，降低企业“过桥”成本；深化与省农业担保集团合作，为农民合作社提供优质金融服务；开展农村土地承包经营权抵押贷款试点，拓宽农业农村融资渠道，着力创新金融平台、创新金融产品，疏导金融“活水”源源不断滋润实体经济，力争在金融创新方面创造焦作经验、走在全省前列。以文化旅游业为坚实基础，做靓服务业品牌。加快十大文化旅游园区项目建设，培育文化旅游经济新增点。完成中心城区旅游发展总体规划，加快旅游服务设施建设，提升城市旅游服务功能。开拓国内外旅游市场，办好云台山国际旅游节。加快云台山旅游股份有限公司上市步伐。推动太极拳申报世界非物质文化遗产。引进社会力量进行市场化运作，谋划筹建太极文化大学，加快开发太极文化产业。依托太极故里、山水焦作、四大怀药等优势，谋划发展大健康产业，推动品牌效应向经济效益转化。

（七）着力农业现代化发展，巩固农业基础地位。按照稳粮增收、提质增效、创新驱动的要求，加快发展现代农业，加快建设美丽乡村，努力使农业更强、农民更富、农村更美。提升农业综合生产能力。新建高标准粮田28万亩。新增农机具5000台，在深松作业、粮食烘干等新型农机推广方面实现新突破。实施现代农业产业化集群培育工程，新增省集群2个，集群销售收入突破600亿元。积极推进农业结构调整。加快四大怀药、绿色蔬菜、食用菌、林产品等特色产业发展，提高生猪、家禽、奶业等产业发展水平，积极发展都市生态农业，加强农畜产品质量安全监管，开展国家农产品质量安全县创建，大力培育农产品品牌，创造农业发展新优势，促进农民增收致富。加强农业基础能力建设。完成山王庄水库等除险加固任务，完成大沙河聂村段等河流治理工程，治理水土流失面积36平方公里，发展节水灌溉面积6万亩。加强农业科技创新，推广农业科技成果。加快气象现代化建设，提高抗灾减灾能力。构建新型农业经营体系。出台支农资金奖励性补贴措施，大力发展专业种养大户、家庭农场、农民合作社、农业产业化龙头企业等新型经营主体，积极培育新型农业社会化服务组织。加快农村土地承包经营权确权登记颁证工作，引导农村土地经营权有序流转，推进农业适度规模经营。新增农民合作社200家，总数达到4000家以上。深入推进新农村建设。坚持“物的新农村”和“人的新农村”建设齐头并进，科学编制新农村建设规划，分类推进新农村综合发展、特色发展。加强农村基础设施建设，新改建农村公路410公里，再解决16万农村群众的饮水安全问题。深入推进美丽乡村建设，改善农村人居环境，争取15%以上的行政村创建成省级示范村，30%以上的行政村创建成省级达标村。

（八）着力生态文明建设，让美丽与发展同行。坚持源头严防、过程严管、后果严惩，加快美丽焦作建设步伐，共建天蓝水清地绿的美好家园。加强生态体系建设。积极创建国家森林城市，高标准推进城市园林绿化，加快北部山区生态治理恢复，完成森林资源培育16.9万亩，努力让森林走进城市、让城市拥抱森林，打造宜居、宜业、宜游的花园城市。强化水生态保护。积极申报国家海绵城市建设试点，大力推进水生态文明城市试点建设，扎实推进城市河道清洁行动，合理开发黄沁河水资源，建设黄沁河生态涵养带，不断改善水生态环境。深入开展环境污染整治。实施燃煤锅炉改造、工业大气污染综合治理等工程，推进汽车尾气、施工扬尘治理和秸秆禁烧等工作，推广应用清洁能源，严厉打击环境违法行为，着力改善空气质量。加强重点流域河流污染治理，推进非工业园区废水排放企业提标改造，加快污水处理厂及配套管网建设。开展重金属污染整治，重点治理皮毛制革、铅蓄电池等行业。加强农村生态环境保护，抓好面源污染治理和土壤污染防治。构建生态建设长效机制。深入推进国家首批资源综合利用双百工程示范基地建设，加快企业节能环保技术改造。做好国家资源枯竭城市转型年度绩效评价工作。完善能源消费总量和重点污染物排放总量预算管理制度，坚持不懈推进节能减排，确保完成“十二五”目标任务。实施节能量、排污权、碳排放、水权交易制度，健全生态环境补偿、环境损害鉴定评估和责任追究等机制，让绿色发展者受益，让损害环境者付出沉重代价。

（九）着力保障改善民生，维护社会和谐稳定。持续加大民生投入，继续实施省、市十项重点民生工程，不断增进人民群众福祉。保持就业稳定增长。深入开展全省统筹城乡就业创业试点市建设，做好高校毕业生、城镇困难人员等重点群体就业工作，大力实施全民技能振兴工程，加大职业技能培训和创业培训力度，以创业带动就业。提高社会保障水平。加强救助体系建设，深入开展对城乡低保、困难群体的临时救助和商业保险医疗救助，健全完善城镇医保按比例报销上不封顶制度和城乡居民基本养老保险制度，加快实施职工大额补充医疗保险即时结算制度，积极推进扶残助残工程，织牢社会保障安全网，兜好民生底线。实施住房公积金惠民工程，开展与银行机构组合贷、网络贷等工作。发展教育文化事业。优化中小学布局，实施农村中小学标准化项目，建成韩公教育园区（一期）等一批城区中小学幼儿园，提升发展特殊教育，逐年提高普通高中学校生均公用经费标准，从秋季起对全日制中职在校生全免学费，努力让每个孩子享受到更好更公平的教育。统筹推进焦作大学、焦作师专升本工作，支持河南理工大学加快发展。实施一批文化惠民工程，做好基层综合文化服务中心建设试点工作，推进市工人文化宫迁建和群艺馆、博物馆新馆规划建设。提升群众健康水平。加快推进市二医院新城区医院、中心血站核酸检测实验室、食品药品检验检测中心等建设。提高出生人口素质，促进人口长期均衡发展。办好第八届国际太极拳交流大赛，创建全省全民健身示范市。落实加快发展养老服务业扶持政策，发挥政府主导作用和社会力量主体作用，推进以居家为基础、社区为依托、机构为支撑、医养相结合的养老服务体系建设。创新社会治理。加强法治宣传教育，完成六五普法任务。加强社会治安综合治理，强化应急管理，建设平安焦作。完善落实安全生产责任体系，切实加强安全监管。推行依法逐级走访制度，排查化解信访隐患。健全完善食品药品监管体系，保障食品药品安全。依法打击和处置非法集资。加强扶贫开发，促进贫困人口稳定脱贫。大力发展志愿者事业。认真做好民族宗教工作。深入开展全国双拥模范城“五连冠”创建，巩固军政军民团结，推进军民融合深度发展。

四、扎实推进政府自身建设

新形势赋予新使命，新常态要有新作为。扎实开展“三严三实”专题教育活动，推动作风建设常态化长效化，持续加强政府自身建设，不断提高政府工作水平。

（一）打造思想解放、创新发展的政府。主动适应新常态、新政策、新要求，摒弃老思维、老套路、老办法，进一步解放思想，牢固树立市场经济观念，不断强化经营城市理念，遵循市场经济规律，善用市场机制手段，靠市场运作项目，靠经营滚动发展，以思维方式创新推动发展方式创新，以创新激发活力、增添动力、加快发展。

（二）打造依法行政、公开透明的政府。把政府工作全面纳入法治轨道，运用法治思维和法治方式深化改革、推动发展、化解矛盾、维护稳定。坚持法定职责必须为、法无授权不可为，严格按照法定权限和程序行使权力、履行职责。依法接受人大及其常委会法律和工作监督，主动接受政协民主监督，严格执行人大及其常委会的决议决定，全力支持配合人大、政协依法履行职能、开展工作，认真办理人大代表建议和政协委员提案，广泛听取民主党派、工商联、无党派人士、人民团体意见，自觉接受群众监督和舆论监督。深入推进政务公开，推进决策公开、执行公开、管理公开、服务公开和结果公开，让人民监督权力，让权力在阳光下运行。

（三）打造担当有为、务实重干的政府。摆脱换挡焦虑、克服本领恐慌，突破守摊子、保位子观念束缚，敢抓敢管、敢闯敢干，以踏石留印的精神、抓铁有痕的劲头，少说多做、说到做到，真抓实干、勇争一流，汇聚经济社会发展正能量。

（四）打造作风优良、服务高效的政府。持续改进工作作风，大力提升服务水平，紧盯发展目标任务，围绕企业发展、项目建设和基层群众，主动作为、优质服务，破解体制机制障碍，提高行政效率效能，不懈怠、不松劲，久久为功、善作善成，用作风大转变、服务大提升，助推焦作发展再上新台阶。

（五）打造廉洁自律、风清气正的政府。坚决落实党风廉政建设“两个责任”，坚决执行廉洁自律各项规定，深入开展反腐败斗争，严肃执纪问责，有腐必惩，有贪必肃。加强岗位权力风险防控机制建设，用制度管权、管事、管人、管钱，扎紧制度笼子，防范权力越线。严格执行中央八项规定和国务院约法三章，坚持倡俭治奢，驰而不息遏制“四风”；加强监督，管好公帑，严肃财经纪律，严控一般性支出，推动公务用车制度改革，确保“三公”经费只减不增，把更多资金用于民生、用于发展。

各位代表！人民赋予重托，奋斗铸就辉煌。让我们紧密团结在以习近平同志为总书记的党中央周围，在省委、省政府和市委的坚强领导下，锐意进取、真抓实干，全面加快经济转型示范市和美丽焦作建设，为创造焦作美好未来而努力奋斗！
